# Phenolic Phytoalexins in Rice: Biological Functions and Biosynthesis

**DOI:** 10.3390/ijms161226152

**Published:** 2015-12-07

**Authors:** Man-Ho Cho, Sang-Won Lee

**Affiliations:** 1 Graduate School of Biotechnology, Kyung Hee University, Yongin 17104, Korea; 2 Department of Plant Molecular Systems Biotechnology & Crop Biotech Institute, Kyung Hee University, Yongin 17104, Korea

**Keywords:** biotic/abiotic stress, phenolic phytoalexins, phenylamide, plant defense mechanism, rice, sakuranetin

## Abstract

Phytoalexins are inducible secondary metabolites possessing antimicrobial activity against phytopathogens. Rice produces a wide array of phytoalexins in response to pathogen attacks and environmental stresses. With few exceptions, most phytoalexins identified in rice are diterpenoid compounds. Until very recently, flavonoid sakuranetin was the only known phenolic phytoalexin in rice. However, recent studies have shown that phenylamides are involved in defense against pathogen attacks in rice. Phenylamides are amine-conjugated phenolic acids that are induced by pathogen infections and abiotic stresses including ultra violet (UV) radiation in rice. Stress-induced phenylamides, such as *N*-*trans*-cinnamoyltryptamine, *N*-*p*-coumaroylserotonin and *N*-cinnamoyltyramine, have been reported to possess antimicrobial activities against rice bacterial and fungal pathogens, an indication of their direct inhibitory roles against invading pathogens. This finding suggests that phenylamides act as phytoalexins in rice and belong to phenolic phytoalexins along with sakuranetin. Phenylamides also have been implicated in cell wall reinforcement for disease resistance and allelopathy of rice. Synthesis of phenolic phytoalexins is stimulated by phytopathogen attacks and abiotic challenges including UV radiation. Accumulating evidence has demonstrated that biosynthetic pathways including the shikimate, phenylpropanoid and arylmonoamine pathways are coordinately activated for phenolic phytoalexin synthesis, and related genes are induced by biotic and abiotic stresses in rice.

## 1. Introduction

Environmental stresses and plant diseases are undoubtedly critical factors in crop production and associated losses [1,2]. Rice is a major staple crop that provides a large portion of human nutrition [3]. In the period of 2001–2003, actual losses of rice production worldwide due to biotic stresses were estimated at 37.4% of the attainable yield, of which pathogens caused 10.8% of the losses [1]. To cope with biotic and abiotic stresses, plants have developed a wide array of defense responses, of which the production of phytoalexins is among their chemical defense repertoire [4,5]. 

Phytoalexins are inducible secondary metabolites possessing antimicrobial activity toward phytopathogens [4,5]. Since the diterpenoids momilactone A and B were identified as phytoalexins in rice leaves infected with blast fungus (*Magnaporthe oryzae*), many diterpenoid phytoalexins, including momilactones, phytocassanes and oryzalexins, have been identified from pathogen-infected rice [6,7,8,9,10,11,12,13]. The flavonoid sakuranetin is highly accumulated in rice leaves in response to blast infection and possesses strong antimicrobial activity against blast fungus, which suggests that it is an important phytoalexin in rice [14]. Although sakuranetin was identified as a phenolic phytoalexin, most rice phytoalexins are diterpenoid compounds, and research efforts have mainly focused on diterpenoid phytoalexins [15,16,17,18,19].

Until very recently, sakuranetin had been considered the only phenolic phytoalexin in rice. However, recent studies have shown that several phenylamides (amine-conjugated phenolic compounds) play a role as defense related agents exhibiting antimicrobial activity against rice pathogens [20,21,22,23]. This observation suggests that, along with sakuranetin, phenylamides are members of phenolic phytoalexins in rice. While the chemical nature and biosynthesis of diterpenoid phytoalexins have been extensively studied and reviewed [13,15,17,18,19], there has been no comprehensive review of phenolic phytoalexins in rice. In this review, we summarize recent progress in rice phenolic phytoalexin research. 

## 2. Phenolic Phytoalexins in Rice

### 2.1. Chemical Nature of Phenolic Phytoalexins in Rice

**Figure 1:**
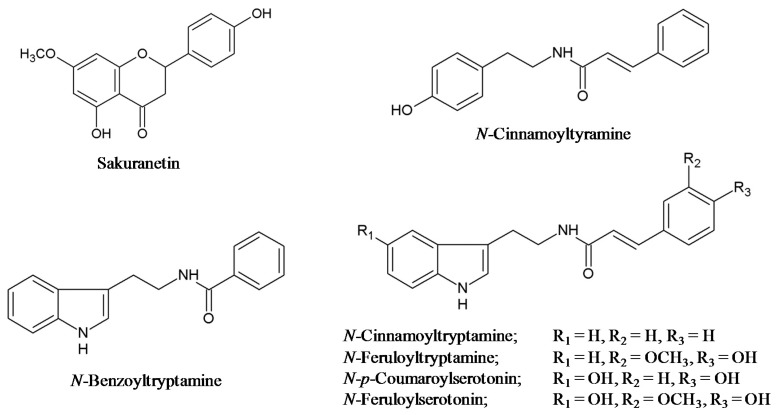
Antimicrobial phenolic compounds, including the methylflavonoid sakuranetin and phenylamides that accumulate in rice in response to biotic and/or abiotic stresses.

Sakuranetin is a well-known phenolic phytoalexin in rice and is a 7-methylated flavanone (Figure 1) [14,22]. In addition to sakuranetin, a group of defense-related phenolic compounds possessing antimicrobial activity was recently identified from pathogen-infected and UV-treated rice leaves [20,21,22,23]. *N-p*-Coumaroylserotonin (CouSer), *N*-feruloyltryptamine (FerTrp) and *N*-feruyolserotonin (FerSer) were identified from rice leaves infected with fungal pathogens, such as blast fungus and rice brown spot fungus *Bipolaris oryzae* (Figure 1) [20,21]. *N*-cinnamoyltyramine (CinTyr), *N*-benzoyltryptamine (BenTrp) and *N*-cinnamoyltryptamine (CinTrp) were identified from UV irradiated rice leaves (Figure 1) [22,23]. These phenolic compounds belong to the phenolics subclass phenylamides, which represent the conjugated form of phenolic acids such as *trans*-cinnamic, *p*-coumaric and ferulic acids with mono- or poly-amines [24,25,26]. Amine moieties found in rice phenylamide phytoalexins include arylmonoamines tyramine, tryptamine and serotonin (Figure 1).

### 2.2. Induced Phenolic Phytoalexin Contents in Rice

Accumulation of a diverse array of diterpenoid phytoalexins in rice is induced by pathogen infections, such as blast-infection [6,7,8,9,10,11,12]. The flavonoid sakuranetin was suggested to be an important rice phytoalexin that is highly accumulated in blast-infected rice leaves compared to diterpenoid phytoalexins [14]. The sakuranetin content in blast-infected rice leaves was reported to be 50.6–100 μg/g fresh leaves (Table 1) [14,27], while the content of momilactones and phytocassanes in blast-infected rice leaves was 8.5–43.2 and 1.9–37.7 μg/g fresh leaves, respectively [12]. Oryzalexins and *ent*-10-oxodepressin contents in blast-infected rice leaves were 2.11–16.8 and 2.48 μg/g fresh leaves, respectively [11,27]. In addition to pathogen attack, UV radiation was reported to be an important elicitor of most rice phytoalexins [7,10,11,23,28]. The sakuranetin content in UV-irradiated rice leaves was reported to range from 22.1–135 μg/g fresh leaves (Table 1), which is comparable to the diterpenoid phytoalexin content in UV-treated rice leaves ranged 1.5–139 μg/g fresh leaves [14,23,27,28]. The sakuranetin content in CuCl_2_ and jasmonic acid (JA) treated rice was reported to be approximately 0.8 and 4 μg/g fresh leaves, respectively (Table 1) [29].

Phenylamides are known to be involved in plant defense by contributing to cell wall reinforcement and providing direct toxic effects against pathogens and insects [26,27,30,31,32,33,34,35]. It was recently reported that the infection of rice brown spot fungus *B. oryzae* results in accumulation of phenylamides, including CouSer, FerTrp and FerSer, in infected rice leaves [20,21]. Rice leaves with decreased accumulation of CouSer and FerSer from treatment with (*S*)-α-(fluoromethyl)tryptophan (*S*-αFMT), an inhibitor of l-tryptophan decarboxylase (TDC), were more severely damaged by *B. oryzae* infection than those not treated with the inhibitor, suggesting that phenylamide metabolites participate in rice defense against *B. oryzae* [21]. The accumulation of FerSer was also reported to be induced in rice leaves by blast infection and methyl jasmonate treatment [20]. Like most rice phytoalexins, the accumulation of phenylamides in rice leaves is induced by UV radiation [22,23]. CouSer, CinTyr, BenTrp and CinTrp were induced by UV irradiation in rice leaves [22,23]. In UV-treated rice leaves, accumulation of phenylamides reached maximum levels one or two days after UV treatment [23]. The phenylamide content in UV-treated rice is comparable with diterpenoid phytoalexin content [23,27,28] with the maximum amounts of UV-induced phenylamides ranging from 32.7–104.2 μg/g fresh leaves (Table 1) [23]. 

**Table 1:** Accumulation of phenolic phytoalexins in pathogen-infected and UV-treated rice leaves.

Phytoalexins		Content ^a^ (μg/g Fresh Weight)	Elicitor	Rice Cultivar	References
Classes	Compounds				
Flavonoid	Sakuranetin	~100	*M. oryzae*	Aichiasahi	[14]
		50.6	*M. oryzae*	Koshihikari	[27]
		135	UV	Koshihikari	[27]
		22.1	UV	Dongjin	[23]
		~0.8	CuCl_2_	Nipponbare	[29]
		~4	JA	Nipponbare	[29]
Phenylamides	CinTyr	49.3	UV	Dongjin	[23]
	BenTrp	65.7	UV	Dongjin	[23]
	CinTrp	32.7	UV	Dongjin	[23]
	CouSer	104.2	UV	Dongjin	[23]
	FerSer	3.2	*B. oryzae*	Nipponbare	[20]

^a^ Maximum phytoalexin content in rice leaves after pathogen infection or UV irradiation.

### 2.3. Antimicrobial Activity and Other Defensive Roles of Phenolic Phytoalexins in Rice

Antimicrobial activities of rice phytoalexins have been investigated with rice pathogens, in particular, blast fungus. Rice diterpenoid phytoalexins inhibit *M. oryzae* spore germination and germ tube growth with half-inhibition concentration (IC_50_) values of 1–35 and 2–103 μg/mL, respectively [8,10,11,12,36]. Sakuranetin was found to exhibit strong inhibitory activity against germ tube growth of blast fungus with an IC_50_ of 5 μg/mL, which is more potent than diterpenoid phytoalexins [14]. Sakuranetin also inhibited mycelial growth of blast fungus with an IC_50_ of 6.44 μg/mL (Table 1) [23]. The IC_50_ of the diterpenoid oryzalexin D against mycelial growth of blast fungus was reported to be 230 μg/mL [37]. In addition to anti-blast fungal activity, a recent study determined that sakuranetin has broad antimicrobial activity against diverse rice fungal and bacterial pathogens [23]. Sakuranetin inhibited mycelial growth of *B. oryzae* and sheath blight fungus *Rhizoctonia solani* with IC_50_ values of 19.05 and 54.04 μg/mL, respectively (Table 2). Growth of rice bacterial pathogens causing bacterial grain rot (*Burkholderia glumae*), blight (*Xanthomonas oryzae* pv. *Oryzae*, *Xoo*), and leaf streak (*X. oryzae* pv. *oryzicola*, *Xoc*) diseases was also reported to be inhibited by sakuranetin with IC_50_ values of 8.22, 19.95 and 2.36 μg/mL, respectively (Table 2), suggesting that it is a broad-spectrum antimicrobial agent against bacterial pathogens as well as fungal pathogens [23].

**Table 2:** Antimicrobial activity of rice phenolic phytoalexins against phytopathogens.

Phytoalexins	Pathogens		Antimicrobial Activity	IC_50_ (μg/mL)	References
Sakuranetin	Fungal	*M. oryzae*	Inhibition of germ tube growth	5	[14]
			Inhibition of mycelial growth	6.44	[23]
		*R. solani*	Inhibition of mycelial growth	54.04	[23]
		*B. oryzae*	Inhibition of mycelial growth	19.05	[23]
	Bacterial	*X. oryzae* pv. *oryzae*	Inhibition of cell growth	19.95	[23]
		*X. oryzae* pv. *oryzicola*	Inhibition of cell growth	2.36	[23]
		*B. glumae*	Inhibition of cell growth	8.22	[23]
CinTyr	Bacterial	*X. oryzae* pv. *oryzae*	Inhibition of cell growth	21.96	[23]
		*X. oryzae* pv. *oryzicola*	Inhibition of cell growth	3.18	[23]
BenTrp	Bacterial	*X. oryzae* pv. *oryzae*	Inhibition of cell growth	34.76	[23]
		*X. oryzae* pv. *oryzicola*	Inhibition of cell growth	3.72	[23]
CinTrp	Fungal	*B. oryzae*	Inhibition of mycelial growth	26.92	[23]
	Bacterial	*X. oryzae* pv. *oryzae*	Inhibition of cell growth	24.34	[23]
		*X. oryzae* pv. *oryzicola*	Inhibition of cell growth	2.45	[23]
		*B. glumae*	Inhibition of cell growth	41.09	[23]
CouSer	Fungal	*Aciculosporium take*	Inhibition of mycelial growth	84	[33]
	Bacteria	*X. oryzae* pv. *oryzicola*	Inhibition of cell growth	54.54	[23]
FerTrp	Fungal	*Fusarium culmorum*	Inhibition of mycelial growth	22	[35]

Phenylamides have been isolated from many plant species and their antimicrobial properties have been reported against bacterial and fungal phytopathogens, suggesting that they play a key role as phytoalexins in plant defense mechanisms [30,31,32,33,34,35]. However, little is known about antimicrobial phenylamides in rice. A recent study showed that stress-induced rice phenylamides possess antimicrobial activities against rice bacterial and fungal pathogens [23]. CinTrp, identified in UV-irradiated rice leaves, inhibited mycelial growth of *B. oryzae* with an IC_50_ of 26.92 μg/mL and exhibited antibacterial activity against *B. glumae*, *Xoo*, and *Xoc*, with IC_50_ values ranging from 2.45–41.09 μg/mL (Table 2) [23]. UV-induced CinTyr and BenTrp were determined to possess antibacterial activities against *Xoo* and *Xoc* (Table 2) [23]. CouSer is induced in rice leaves by both UV-irradiation and *B. oryzae* infection and inhibits the growth of *Xoc* with an IC_50_ of 54.54 μg/mL (Table 2) [23]. CouSer was also identified as a bamboo plant phytoalexin, showing antimicrobial activity against witches’ broom fungus *Aciculosporium take* with an IC_50_ value of 84 μg/mL (Table 2) [33]. FerTrp isolated from the roots of *Allium* species was reported to have antifungal activity against *Fusarium culmorum* [35]. Phytopathogen and abiotic stress-induced synthesis and antimicrobial properties of rice phenylamides suggest that they are a new class of rice phytoalexins comprising phenolic phytoalexins such as sakuranetin. 

In addition to their function as antimicrobial agents, a number of studies have suggested that phytoalexins play diverse roles in plant defense responses to biotic and abiotic stresses [5]. Diterpenoid momilactones A and B were isolated as allelochemicals from rice seed husk and were later identified as phytoalexins because of their blast-induced biosynthesis and antifungal activity [6,38]. Biosyntheses of momilactones A and B and most rice diterpenoid phytoalexins were also reported to be induced by UV exposure [7,10,11,23,28]. The flavonoid phytoalexin sakuranetin was induced by UV irradiation as a major flavonoid aglycone in UV-treated rice leaves [14,22,23]. Phenolics, such as flavonoids and phenolic acids, are well known to act as sunscreen against harmful UV and scavengers of reactive oxygen species (ROS) in plants [39,40,41]. Due to the amine and phenolic acid moieties, phenylamides act as antioxidants that scavenge free radicals [26]. Accumulation of phenylamides, such as ferulylputrescine and cinnamoylputrescine, were reported to be associated with the formation of free radicals in tobacco and beans under excess water and high temperature stresses, respectively [42]. FerTrp and CinTyr were found to exhibit radical scavenging activity [43]. In rice leaves, phenylamide phytoalexins were produced upon UV exposure [22,23]. The accumulation of sakuranetin and phenylamide phytoalexins in UV-irradiated rice leaves suggests a defensive role of rice phytoalexins in UV-induced oxidative stress [22].

As a physical barrier, the cell wall is important in plant defense against biotic and abiotic stresses. During the defense response to pathogen attacks and wounding, the cell wall is reinforced by deposition of cell wall biopolymers, such as callose and lignin, to prevent the entrance and propagation of invading pathogens [5,44,45]. Wound-induced synthesis of phenylamides, such as feruloyltyramine and *p*-coumaroyltyramine, and their deposition in the cell wall are well known in wounded potato tissues, suggesting that they contribute to defensive biopolymers [46,47,48]. In rice, tryptamine, serotonin and tryptamine derived phenylamides induced by *B. oryza* infection were reported to be deposited in the cell wall of lesion tissues [20,21]. Treatment with the tryptamine biosynthesis inhibitor *S*-αFMT suppressed the accumulation of tryptamine and its phenolic-conjugates and decreased the deposition of these materials in lesion tissues, which resulted in inhibitor treated leaves being susceptible to *B. oryzae* infection [21]. This finding indicates that pathogen-induced rice phenylamides are involved in the defense response through reinforcement of the cell wall near infection sites in addition to their phytoalexin function. 

Allelochemicals are produced in plant tissues and are released into the environment, suppressing the growth and establishment of neighboring plants [49,50,51]. Allelopathic properties of diterpenoid phytoalexins, such as momilactones A and B, have been well established in rice [37,52,53]. Momilactones are secreted from rice roots into the environment and play a role as allelochemicals [52,53]. Phenolic acids, such as *p*-coumaric acid, ferulic acid and caffeic acid, were also isolated from the roots of allelopathic rice cultivars and were identified as allelochemicals [54,55,56]. The phenolic acid moieties in phenylamide phytoalexins suggest that they likely act as allelochemicals in rice. In addition to UV and pathogen induced accumulation of phenylamide phytoalexins in rice leaves, CinTrp and BenTrp were isolated from rice roots without external stimulus [57]. A recent study also demonstrated that the phenylamide CinTyr isolated from rice acts as an allelochemical that inhibited root and hypocotyl growth of cress, barnyard grass, and red sprangletop [58]. This result suggests that phenylamide phytoalexins are potential allelochemicals in rice.

## 3. Biosynthesis of Rice Phenolic Phytoalexins

During stress-induced production of sakuranetin and phenylamide phytoalexins, a series of metabolic pathways are potentially activated in rice tissues. The biosynthetic pathways implicated in rice phenolic phytoalexin synthesis include the shikimate pathway for aromatic l-amino acids (AAs) and the phenylpropanoid pathway for phenolic acid moieties in phenylamides and sakuranetin [22,39,40,59,60,61]. In plants, most genes for these pathway enzymes exist as multigene families, of which a set of genes are induced and implicated in biotic and abiotic stress-triggered synthesis of phenolic phytoalexins in rice [22,59,62,63]. Recently, functional studies of genes involved in phenolic phytoalexin synthesis have been performed with rice. Here, we summarize the induced biosynthetic pathways and genes for rice phenolic phytoalexin biosynthesis in response to various stresses, in particular UV irradiation and phytopathogen attack.

### 3.1. Shikimate and Phenylalanine Biosynthetic Pathway

**Figure 2:**
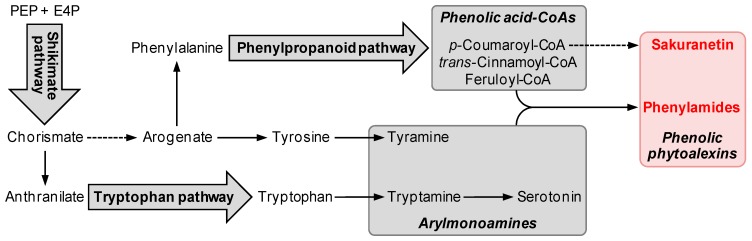
Biotic and abiotic stress-induced metabolic pathways for phenolic phytoalexin biosynthesis in rice. The shikimate, phenylpropanoid and tryptophan pathways are coordinately activated by biotic and abiotic stresses to synthesize phenolic phytoalexins in rice. Phenolic acid-CoAs, such as *p*-coumaroyl-, *trans*-cinnamoyl- and feruloyl-CoAs, serve as intermediates in the formation of sakuranetin and phenylamide phytoalexins. Arylmonoamines, such as tryptamine, tyramine and serotonin, are conjugated with phenolic acid-CoAs to form phenylamide phytoalexins. Dashed arrows indicate multiple enzymatic steps. PEP; phosphoenol pyruvate, E4P; erythrose 4-phosphate.

Aromatic AAs are building blocks for protein synthesis and serve as common precursors for plant secondary metabolites, such as phenolics and nitrogen containing compounds [60]. The shikimate pathway, an early biosynthetic pathway for aromatic AAs, is activated in plants under stress conditions (Figure 2) [22,60,64]. The shikimate pathway synthesizes chorismate, a common intermediate for aromatic AAs, from phosphoenol pyruvate and erythrose 4-phosphate [60]. Activation of the shikimate pathway by pathogen attack was demonstrated with the metabolomic analysis of *M. oryzae* infected rice leaves [64]. A recent transcriptomic analysis also showed that shikimate pathway genes were induced in rice leaves in response to UV radiation [22]. A set of shikimate pathway genes including 3-deoxy-d-arabino-heptulosonate 7-phosphate synthase (*DAHPS*, Os07g42960), 3-dehydroquinate synthase (*DHQS*, Os09g36800), 3-dehydroquinate dehydratase/shikimate dehydrogenase (*DHQDT/SDH*, Os01g27750 and Os12g34874), shikimate kinase (*SK*, Os06g12150 and Os02g51410), and chorismate synthase (*CS*, Os03g14990), are immediately induced by UV treatment prior to the accumulation of sakuranetin and phenylamide phytoalexins, which implies the possible involvement of these genes in phytoalexin biosynthesis in rice (Table 3) [22]. Chorismate is subsequently converted to l-phenylalanine (Phe) and l-tyrosine (Tyr) by chorismate mutase (CM), prephenate aminotransferase (PAT), and arogenate dehydratase (ADT) or arogenate dehydrogenase [60]. Induction of *CM* (Os01g55870) and *ADT* (Os10g37980) were observed in rice leaves in response to UV treatment (Table 3) [22]. Direct evidence for a phytoalexin synthesis-related function of shikimate pathway genes and Phe and Tyr biosynthetic genes in rice has not yet been reported.

### 3.2. Phenylpropanoid Pathway

In plants, phenolic compounds, such as lignin, soluble phenolics and flavonoids, are primarily derived from Phe through the phenylpropanoid pathway [39,40,59,61]. Phe is deaminated to form *trans*-cinnamate by phenylalanine ammonia lyase (PAL). *trans*-Cinnamate is further converted to a diverse array of phenylpropanoid metabolites by the concerted action of phenylpropanoid pathway enzymes, including cinnamate 4-hydroxylase (C4H), *p*-coumarate 3-hydroxylase (C3H), 4-coumarate: CoA ligase (4CL) and caffeate *O*-methyltransferase (COMT). Phenylpropanoid metabolites serve as precursors for sakuranetin and phenolic moieties in phenylamide phytoalexins (Figure 2) [22,26,61,65,66].

PAL is a key phenylpropanoid pathway enzyme, which participates in the biosynthesis of the defense-related hormone salicylic acid and phenolic compounds including phenolic phytoalexins [22,67]. *PAL* genes (*OsPAL*s) in rice consist of a large gene family with 11 members in the Michigan State University (MSU) rice genome database (version 7.0) released from the MSU Rice Genome Annotation Project [62,63]. A knockout mutant of *OsPAL6* (Os02g41680) showed increased susceptibility to root-invaded *M. oryzae* and the almost complete disappearance of sakuranetin in the infected rice roots, which suggests a role of this pathway in disease-resistance to blast fungus and sakuranetin biosynthesis [67]. Induced expression of *OsPAL6* was observed in rice roots infected with *M. oryzae* but not in the infected leaves. In rice leaves, two *OsPAL*s (Os02g41630 and Os02g41650) were induced by *M. oryzae* infection [67]. Induced expression of these *OsPAL*s was also observed in UV-treated rice leaves along with accumulation of sakuranetin and phenylamide phytoalexins, which suggest their role in phytoalexin biosynthesis in rice leaves [22]. Although their function in phytoalexin production was not examined, an involvement of *OsPAL*s in the defense response against pathogens has been suggested. The deletion mutation of an *OsPAL4* (Os02g41680) caused increased susceptibility to fungal and bacterial pathogens [68]. Induction of two *OsPAL*s [*PAL07* (Os04g43800) and *PAL04* (Os05g35290)] was reported in rice suspension culture treated with cell wall hydrolyzates prepared from *M. oryzae* [69]. 

Hydroxy-cinnamates, such as *p*-coumarate and caffeate, are formed from *trans*-cinnamate by hydroxylases, such as C4H and C3H. In rice, functional *C4H* and *C3H* genes have yet to be identified. Four cytochrome P450s similar to C4H are found in the MSU rice genome database [69]. UV-induced expression of a cytochrome P450 gene (Os2g26770) similar to C4H was observed in the microarray analysis of UV-treated rice leaves (Table 3) [22]. COMTs and caffeoyl-CoA *O*-methyltransferases (CCoAOMTs) participate in the formation of methylated phenolic acid-CoAs, such as feruloyl-CoA [59,61]. Transcriptomic analysis of UV-treated rice leaves showed that four putative OMTs (Os11g19840, Os09g17560, Os08g06100 and Os04g01470) similar to flavonoid OMTs and COMTs are up-regulated in response to UV irradiation prior to accumulation of sakuranetin and phenylamide phytoalexins, which suggests their possible involvement in phenolic phytoalexin synthesis in rice [22].

Phenolic acid-CoAs, such as *p*-coumaroyl-CoA and feruloyl-CoA, are key branch points in phenolic metabolism. They serve as activated intermediates for flavonoid and phenylamide biosynthesis. 4CLs catalyze the ligation of CoA to phenolic acids. Five Os4CLs (Os4CL1-5) were characterized, and their roles in lignin synthesis and phenolic metabolism were investigated in rice [70,71]. In addition, 10 4CL-like genes exist in the MSU rice genome database [62]. *Os4CL2* (Os02g46970) is specifically expressed in the anther and is strongly induced by UV irradiation, which suggests its role in flavonoid biosynthesis [71]. In UV-treated rice leaves, activation of a 4CL-like gene (Os03g04000) expression was accompanied by an accumulation of phenolic phytoalexins, which suggests its possible involvement in phytoalexin synthesis in rice (Table 3) [22].

### 3.3. Sakuranetin Biosynthesis

Sakuranetin is the 7-methylated form of the flavanone naringenin (Figure 1) [14,22]. *p*-Coumaroyl-CoA is condensed with three molecules of malonyl-CoA to naringenin chalcone by chalcone synthase (CHS), which is then converted to the flavanone naringenin by chalcone isomerase (CHI) [39,40,59]. Thirty genes similar to CHS and seven CHI-like genes were found in the MSU rice genome database [62]. Although a recent transcriptomic analysis of UV-treated rice leaves showed activation of three *OsCHS*s (Os04g01354, Os07g31770 and Os11g32650) and two *OsCHI*s (Os02g02370 and Os11g02440) along with the accumulation of sakuranetin [22], further research is required to identify *OsCHS* and *OsCHI* genes involved in sakuranetin formation. The final step of sakuranetin formation is a methylation step of the 7-OH of the flavanone naringenin. Naringenin *O*-methyltransferase (OsNOMT) for sakuranetin synthesis was purified from UV-irradiated *oscomt1* rice leaves, and the corresponding gene (Os12g13800) was identified (Table 3) [29]. Expression of *OsNOMT* was induced by JA and UV treatment in rice leaves prior to sakuranetin accumulation [22,29]. Possible involvement of UV-induced OMTs closely related to flavonoid OMTs in sakuranetin synthesis was suggested by phytochemical and transcriptomic analyses of UV-irradiated rice leaves (Table 3) [22]; however, their definitive role in phenolic phytoalexin biosynthesis in rice needs to be further studied.

### 3.4. Arylmonoamine Biosynthesis and Its Conjugation with Phenolic Acid-CoAs

Phenylamides are formed by the conjugation of arylamines and phenolic acids [22,26,65]. Arylmonoamines found in rice phenylamide phytoalexins are tyramine, tryptamine, and its derivative serotonin (Figure 1) [20,21,22,23]. Tyramine and tryptamine are derived from Tyr and l-tryptophan (Trp), respectively. Tyr biosynthesis mostly shares the phenylalanine biosynthetic pathway and is induced in rice leaves in response to UV treatment [22,60]. The Trp biosynthetic pathway from chorismate was reported to be activated by *B. oryzae* and *M. oryzae* infection and UV irradiation [20,22].

Chorismate is converted to anthranilate by anthranilate synthase (AS), which is a heteromer composed of α (ASα) and β (ASβ) subunits in plants [60]. The rice genome contains two ASα (*OsASA1* and *OsASA2*) and two ASβ (*OsASB1* and *OsASB2*) genes. In rice leaves, *OsASA2* (Os03g15780) was induced by chitin elicitor treatment and infection with phytopathogens, such as *B. oryzae*, *Xoo*, and *M. oryzae*, which was accompanied by an increase in intermediates for tryptophan biosynthesis [20,72,73]. Both *OsASB1* (Os04g38950) and *OsASB2* (Os03g50880) were induced in rice by UV treatment and infection with pathogens, including *B. oryzae* and *Xoo* [20,22,72,73]. Induction of *OsASB2* was also observed in the rice *spotted leaf 5* (*spl5*) mutant, which showed enhanced resistance to pathogens [74]. 

Anthranilate is further converted to Trp by the serial action of anthranilate phosphoribosyltransferase (APT), phosphoanthranilate isomerase (PAI), indole-3-glycerol phosphate synthase (IGPS), and Trp synthase (TS) [60]. *APT* (Os03g03450) and *PAI* (Os02g16630) expressions were found to be induced by pathogen attack [73]. Induced expression of an *IGPS* gene (Os09g08130) was observed in UV-treated rice leaves and in the rice *spl5* mutant [22,74]. TS is composed of α (TSα) and β (TSβ) subunits. The transcript level of *TSA* (Os07g08430) and TSα protein abundance were increased in UV-treated and pathogen-infected rice leaves [22,73,75]. *TSA* expression was also increased in the rice *spl5* mutant [74]. *TSB1* (Os08g04180) was induced by UV treatment and pathogen attack in rice leaves [22,73]. The induction of *TSB2* (Os06g42560) was also observed in UV-treated rice leaves [22]. 

Trp decarboxylase (TDC) and Tyr decarboxylase (TYDC), which belong to a family of aromatic AA decarboxylases (AADCs), catalyze the conversion of tryptophan and tyrosine to the corresponding arylmonoamines, tryptamine and tyramine, respectively, [76]. Induced TDC activity by *M. oryzae* infection was reported in *sekiguchi lesion* (*sl*) mutant rice [77]. The *AADC* gene (Os08g04540) was induced in UV treated rice leaves [22]. This *AAD*C was heterologously expressed in *E. coli*, and the recombinant enzyme exhibited TDC activity [78]. Over-expression of this gene led to increased tryptamine and serotonin levels in transgenic rice plants [78].

Serotonin, 5-hydroxytryptamine, is formed from tryptamine by tryptamine 5-hydroxylase (T5H) [73,79]. Rice sekiguchi lesion (*sl*) mutants were observed to accumulate tryptamine, and the *SL* gene was identified as the cytochrome P450 monooxygenase gene (Os12g16720) encoding T5H [79]. Expression of *SL* was induced by chitin elicitation and *M. oryzae* infection [79]. This *T5H* gene was also induced in UV or cadmium-treated rice leaves [22,80]. This result suggests that the T5H encoding *SL* gene is involved in serotonin formation during stress responses.

**Table 3:** Stress-induced genes tentatively involved in phenolic phytoalexin biosynthesis in rice.

Pathways/Enzyme	No. ^a^	Gene Name ^b^ (or Locus ID ^c^)	Elicitors (or Evidence)	References
*Shikimate pathway*				
*DAHPS*	7	Os07g42960	UV	[22]
*DHQS*	1	Os09g36800	UV	[22]
*DHQDT/SDH*	6	Os01g27750, Os12g34874	UV	[22]
*SK*	10	Os06g12150, Os02g51410	UV	[22]
*CS*	1	Os03g14990	UV	[22]
*CM*	5	Os01g55870	UV	[22]
*ADT*	12	Os10g37980	UV	[22]
*Phenylpropanoid pathway*				
*PAL*	11	*OsPAL6* (Os02g41680)	*M. oryzae*	[67]
		*OsPAL1* (Os02g41630), *OsPAL8* (Os02g41650)	*M. oryzae*, UV	[22,67]
		*OsPAL4* (Os02g41680)	Mutation causes disease susceptibility	[68]
		*PAL07* (Os04g43800), *PAL04* (Os05g35290)	Cell hydrolyzates from *M. oryzae*	[69]
*C4H*	4	Os2g26770	UV	[22]
*4CL*	15	*Os4CL2* (Os02g46970)	UV	[71]
		Os03g04000	UV	[22]
*COMT*		Os11g19840, Os09g17560, Os08g06100, Os04g01470	UV	[22]
*Sakuranetin biosynthesis*				
*CHS*	30	Os04g01354, Os07g31770, Os11g32650	UV	[22]
*CHI*	7	Os02g02370, Os11g02440	UV	[22]
*NOMT*		*OsNOMT* (Os12g13800)	UV, JA	[22,29]
*Tryptophan pathway*				
*ASα*	2	*OsASA2* (Os03g15780)	*B. oryzae*, chitin, *Xoo*, *M. oryzae*	[20,72,73]
*ASβ*	2	*OsASB1* (Os04g38950), *OsASB2* (Os03g50880)	UV, *B. oryzae*, *Xoo*, *M. oryzae*	[20,23,73]
*APT*	2	Os03g03450	*Xoo*, *M. oryzae*	[73]
*PAI*	1	Os02g16630	*Xoo*, *M. oryzae*	[73]
*IGPS*	3	Os09g08130	UV, induced in the *spl5* mutant	[22,74]
*TSα*	5	*TSA* (Os07g08430)	UV, induced in the *spl5* mutant, *Xoo*, *M. oryzae*	[22,73,74,75]
*TSβ*	2	*TSB1* (Os08g04180)	UV, *Xoo*, *M. oryzae*	[22,73]
**		*TSB2* (Os06g42560)	UV	[22]
*Phenylamide biosynthesis*				
*AADC*	7	Os08g04540	UV, *M. oryzae*	[22,77]
*T5H*		*SL* (Os12g16720)	*M. oryzae*, chitin, CdCl_2_, UV	[22,79,80]
*Acyltransferase*		Os11g42370, Os04g09260, Os09g37180, Os04g56900, Os01g42880, Os02g39850, Os01g09010, Os07g36560	UV	[22]

^a^ Number of annotated genes in each gene family; ^b^ Name of gene in references; ^c^ Gene locus identifiers in the MSU rice genome database (version 7.0) [62].

Phenylamides are formed by the conjugation of arylmonoamines with phenolic acid-CoAs catalyzed by acyltransferases such as BAHD acyltransferases and tyramine: *N*-hydroxycinnamoyl transferase-like enzymes [22,65,81]. Little is known about functional acyltransferases involved in phenylamide phytoalexin biosynthesis in rice. Microarray analysis of UV-treated rice leaves showed that several acyltransferases belonging to clades VI and V of the BAHD acyltransferase family are immediately induced in response to UV treatment along with the induction of arylamine and phenolic acid-CoA biosynthetic genes (Table 3) [22]. The clades VI and V of the BAHD family contain *N*-acyltransferases [81]. Thus, the UV-induced BAHD acyltransferases have been suggested to be involved in phenylamide phytoalexin biosynthesis in rice [22]. A definitive role of stress-induced BAHD acyltransferase genes in phenylamide phytoalexin synthesis requires further study in rice.

## 4. Conclusions

It has long been known that rice phytoalexins are mostly diterpenoid compounds. Recent studies, however, have shown that phenolic compounds play an important role in disease resistance of rice as phytoalexins, which include a range of phenylamides and sakuranetin. The phenolic phytoalexins are also implicated in rice defense against biotic and abiotic stresses through the reinforcement of cell walls and scavenging ROS, as well as exhibiting an allelopathic property. In addition to their biological functions in plants, rice phytoalexins show a diverse range of health beneficial properties. Sakuranetin exhibits antibiotic activity to *Helicobacter pylori* and allergy preventive activity [82,83]. Phenylamide phytoalexins, such as CinTrp and CouSer, are reported to have antimicrobial properties against pathogenic bacteria, as well as antiinflammatory and antiatherogenic effects [43,84,85]. Thus, more research is required to clearly understand the biological roles and biosynthetic rotes of phenolic phytoalexins in rice, as well as for their biotechnological applications.
